# High impact chronic pain in children: exploration of associated characteristics and clinical factors in children and parents

**DOI:** 10.3389/fpain.2026.1744974

**Published:** 2026-02-13

**Authors:** Ulla Caverius, Marcelo Rivano Fischer, Jan Lexell, Sophia Åkerblom

**Affiliations:** 1 Department of Health Sciences, Rehabilitation Medicine, Lund University, Lund, Sweden; 2 Department of Neurosurgery and Pain Rehabilitation, Skane University Hospital, Lund, Sweden; 3 Department of Rehabilitation Medicine, Angelholm Hospital, Ängelholm, Sweden

**Keywords:** adolescents, associated factors, children, functioning, high impact chronic pain, parents

## Abstract

**Background:**

Chronic pain in children can significantly impact daily functioning. While interdisciplinary pain rehabilitation is the recommended treatment, it is resource-intensive and limited in availability. Therefore, children with more complex symptoms, classified as High Impact Chronic Pain (HICP), need to be prioritized for interdisciplinary pain rehabilitation. However, HICP in children has been incompletely defined in previous literature, and associated factors remain underexplored. This study aimed to explore a broader biopsychosocial definition of HICP in children and to investigate characteristics and clinical factors in children and parents associated with HICP.

**Method:**

This exploratory retrospective cross-sectional registry study included 484 children with chronic pain and their parents, referred to a tertiary pediatric pain clinic in Sweden. HICP was defined using five variables: pain intensity, pain interference, overall well-being, insomnia, and school absence. Children who met thresholds in at least three of these variables were classified as having HICP. Multivariable logistic regression models were used to identify factors in children and parents associated with HICP.

**Results:**

A total of 60% of the participants met the criteria for HICP. Variables significantly associated with HICP included symptoms of depression (OR 1.10, *p* = 0.001) and psychological inflexibility (OR 1.06, *p* = 0.001) in children, as well as pain reactivity in parents (OR 1.05, *p* = 0.018).

**Conclusion:**

Psychiatric comorbidity and behavioral aspects, including psychological inflexibility and parental pain reactivity, play key roles in pediatric HICP and should be considered when assessing and planning interventions. Addressing these factors may improve treatment outcomes and reduce long-term challenges.

## 1 Introduction

Chronic pain in children and adolescents (hereafter called children) is common, but the group is heterogeneous. Many children appear to cope well with chronic pain in daily life, wereas for others, pain is associated with high impact on a wide range of areas such as school attendance, physical and social activities, family functioning and psychological well-being (1–3). The latter has been referred to as “high impact chronic pain” (HICP) (4, 5). To assess functioning in chronic pain, a biopsychosocial approach is recommended in international studies and guidelines, emphasizing the interplay among biological, psychological, and social factors (3, 6, 7). This approach also forms the basis of interdisciplinary pain rehabilitation (IPR), grounded in a cognitive behavioral framework, which is the recommended treatment strategy for children with chronic pain (3).

IPR is typically provided in specialized pediatric pain clinics. However, access remains limited, and long geographical distances further restrict availability in countries such as Sweden (8, 9). This may lead to inadequate care and increase the risk that the pain persists into adulthood with limitations in physical and social activities, vocational ability and mental health (10–15). IPR is also resource-intensive and expensive in terms of personnel and treatment time (16). Consequently, children with more severe and complex symptoms, classified as having HICP, should be prioritized for IPR, and their specific needs and contributing factors must be clearly identified.

Previous studies on HICP in adults and children have mostly relied on pain intensity and pain interference for classification purposes (4, 5, 17–20), leaving an opportunity to adopt a broader biopsychosocial perspective that more fully captures the complexity of symptoms in children. In addition, the characteristics or clinical factors that contribute to HICP are not well investigated in children (4, 21, 22). For instance, studies including parental or behavioral aspects are lacking. These aspects need to be investigated in greater detail to enable tailored rehabilitation going forward.

To remedy the identified research gaps, the first aim of this study is to define HICP in children from a broader biopsychosocial perspective covering physical, psychological and social aspects, and including core outcome variables for pediatric pain studies (23, 24). The second aim is to investigate characteristics and clinical factors in children and their parents as potential factors associated with HICP as indicated by pain severity, pain interference, overall well-being, sleep disturbance and school absence.

## 2 Material and methods

### 2.1 Research design

This is an exploratory retrospective cross-sectional registry study of children with chronic pain and their parents at their first appointment at a tertiary pain clinic located in Sweden. The study is part of a larger research project based on a clinical registry, previously described in detail (25). The study follows the Strengthening the Reporting of Observational studies in Epidemiology guidelines (STROBE) and contains the necessary items according to the STROBE checklist to properly report an observational study (26).

### 2.2 Participants and data collection

All children 10 to 18 years of age and their parents (*N* = 484) who were assessed at a Specialized Pediatric Pain Clinic (SPPC) for chronic pain at Skåne University Hospital, Sweden, between 2013 and 2021 and who approved participation in the registry were included in the study. Children were usually referred from pediatricians, child psychiatrists or primary care physicians and had previously undergone an assessment of any underlying cause/diagnosis for their pain. All children had chronic pain as defined by IASP, i.e., pain that persists or recurs for longer than 3 months. At least one parent attended the first appointment together with their child and answered the routine parental questionnaires.

Before the first assessment, children and their parents filled in the self-report questionnaires and background data which they sent in or brought with them to the clinic. Additional data were obtained from the interviews during the interdisciplinary assessment and the medical records, and kept together with the self-report data in the clinic's registry.

Data on physical, psychological and social functioning as well as sociodemographic data, pain characteristics, and symptoms of psychiatric comorbidities for the present study, were obtained from the registry.

There were only 26 children under 10 years of age in the registry, and these were not included because most questionnaires are not validated for younger children and the parents routinely report the child's functioning by proxy. Anyone not fluent in Swedish was offered an interpreter.

### 2.3 Assessments

#### 2.3.1 High impact chronic pain

High impact chronic pain (HICP) was captured using five indicators: pain intensity (Numeric Rating Scale, NRS), pain interference (Pain Interference Index, PII), overall well-being (Pediatric Quality of Life Inventory total score, PedsQL tss, including physical, emotional, and role functioning), school absence (%), and sleep disturbance (Insomnia Severity Index, ISI). HICP was defined by meeting at least three of the following five criteria: NRS ≥ 5/10 (27); PII ≥ 18/36; PedsQL total score <70% (28); school absence ≥ 20% (27); ISI ≥ 9/28 (29, 30). Children meeting two or fewer of these criteria were classified as having low impact chronic pain (LICP). The domains covered all recommended mandatory and important outcome domains for pediatric pain research on chronic pain that are relevant for baseline assessment (24). School absence due to pain is a major concern for many children with chronic pain, as it is often associated with declines in academic performance and broader aspects of school functioning (31, 32). Therefore, this domain was included in the definition of functioning.

### 2.4 Measures

#### 2.4.1 Variables defining HICP

*Numerical Rating Scale* (NRS) was used to assess pain intensity (33, 34). The children were asked to rate the pain at the moment. NRS is graded 0 to 10, with 0 representing “no pain” and 10 “worst pain imaginable”. A pain score of ≥5 was considered high pain severity (27).

The *Pain Interference Index* (PII) was used to assess the impact of pain on daily life. Six questions are answered about how the pain during the past two weeks has affected various aspects of daily life such as physical activity, school, friends, leisure activities, sleep and mood. The maximum score is 36 and each answer is graded from 0 (not at all) to 6 (completely). The scale has shown good psychometric properties in studies of both children and adults with chronic pain in both Swedish and international studies (35–37). Internal consistency of items in the PII, determined by Cronbach's α, was 0.86 in a Swedish study of children with chronic pain aged 7 and 18 years (35). As no established cut-off scores for HICP are currently available, we determined the threshold for high impact in this study based on the normal distribution of PII. Accordingly, a cut-off value of 18 was applied.

The *Pediatric Quality of Life Inventory* (PedsQL™ 4.0 Generic Core Scales) was used to assess overall well-being. PedsQL encompasses 4 subscales: physical functioning (8 items), emotional functioning (5 items), social functioning (5 items) and school functioning (5 items). Higher scores indicate better health-related quality of life (HRQoL). For children 8 years and older a cut-off score of 78 indicates special health care needs, 76 indicates chronic conditions and 70 indicates major chronic conditions (28). For this study the cut-off score for high impact was set to <70. PedsQL self-report has shown high validity and reliability in an international study of children 5 to16 years old and in a Swedish study of schoolchildren, with a Cronbach's α of >0.7 for all subscales and close to 0.9 for the total score scale (38, 39).

*School absence* was recorded by the number of school days missed as reported by the child's parents, which has previously been shown to correlate well with official school attendance records (31). Late arrival to school or leaving school early due to pain was recorded as a half day missed. For HICP classification purposes, a cut-off of ≥20% school absence per week attributable solely to pain was applied. An earlier study has suggested similar cut-off scores for high pain related limitation (27).

The *Insomnia Severity Index* (ISI) was used to assess sleep disturbances. The ISI consists of 7 items rated from 0 (not a problem/not at all) to 4 (very much) and covers different aspects of sleep disturbances such as falling asleep, nocturnal and early morning awakenings, satisfaction with sleep and impaired daily functioning due to sleep difficulties. Maximum score is 28 and higher scores indicate greater impact on sleep. Cut-off for adults are set to: 0–7 no clinically significant insomnia; 8–14 subthreshold insomnia; 15–21 moderate clinical insomnia; 22–28 severe clinical insomnia (40). The ISI has been shown to be a reliable and validated instrument for detecting insomnia and for evaluating treatment effects and has been translated into different languages (41). A Chinese study validated ISI for adolescents 12 to 19 years old in a general population and used a cut-off score of 9 (29). This cut-off has later shown satisfactory psychometric properties in a Swedish study of children 10 to 18 years old with chronic pain, with measured internal consistency, Cronbach's α of 0.88 (30). Hence, a cut-off of 9 was used for HICP in this study.

#### 2.4.2 Associated variables

##### 2.4.2.1 Sociodemographics

Sex and age were registered for all participants*.* Marital status, highest education, and employment rate were also recorded for parents and dichotomized.

##### 2.4.2.2 Pain characteristics

Children marked on a pain chart the parts of the body where they were in pain (42). The location(s) that hurt the most were marked with an “X”, and the number of pain locations was dichotomized into <3 or ≥3. During the assessment, parents were asked whether they experienced any ongoing long-term pain and, if so, how it impacted their ability to work. Work ability was dichotomized into working full-/part time or being on sick leave, disability pension, old-age pension or unemployed.

##### 2.4.2.3 Psychological and coping/behavioral factors

*Center for Epidemiological Studies—Depression Child* (CES-DC) was used to assess symptoms of depression in children over the past week. CES-DC is a 20-item scale with a maximum score of 60. A cut-off score of 24 has been identified in a Swedish study as sufficiently specific for detecting depressive disorders in adolescents. The scale has been validated both internationally and within Sweden (43, 44).

*Pain Reactivity Scale-child* (PRS-c) was used to measure psychological reactivity such as pain-related worrying in children. Five statements about pain-related concerns and emotional reactions are graded from 0 (not at all) to 6 (always). The maximum score is 30 and higher scores indicate more discomfort. Several Swedish studies have used PRS for research on chronic pain in children and adults and PRS has satisfactory psychometric properties (37, 45, 46).

*Pain Reactivity Scale-Parent* (PRS-p) was used to report the parent's worry about the child's pain. The five questions in the questionnaire are the same as in the child questionnaire, but with the parent's point of view, such as “How often do you worry about your child's pain?”. The scoring is the same as for the PRS child form, with a maximum score of 30 (37, 45).

*Psychological Inflexibility in Pain Scale* (PIPS) was used to assess psychological inflexibility in children, with two subscales assessing pain avoidance (8 items) and cognitive fusion (4 items). These are rated from 1 (never true) to 7 (always true), with a maximum score of 84. High scores indicate higher psychological inflexibility. PIPS has shown acceptable validity and reliability for adults (47–49) and for children (50).

*Parental Psychological Flexibility Questionnaire* (PPFQ-10) was used to capture parental responses to the child's pain in terms of psychological flexibility. Psychological flexibility is defined as the parent's willingness to experience distress related to the child's pain in the service of valued behaviour. PPFQ-10 is a short version of PPFQ, which are both valid and reliable tools (51–53). The internal consistency for PPFQ-10 in a Swedish sample of parents of children with chronic pain measured a Cronbach's α of 0.86 (53). Questions are rated from 0 (never true) to 6 (always true). Maximum score of PPFQ-10 is 60 and higher scores indicate greater psychological flexibility (52–54).

### 2.5 Ethical considerations

Written information was sent to all families, who without exception, gave their consent to the storage of information and the use of data for future research. The study was approved by the Swedish Ethical Review Authority 2021-07-26 (No. 2021-02709). Throughout the study the Declaration of Helsinki for research on humans was followed.

### 2.6 Data analyses

A binary variable for HICP was constructed. Children who had at least 3 of 5 of the following items were considered to have HICP: Pain intensity (NRS present) ≥5/10 (27), Pain interference (PII) ≥18/36 (see Measures section), Quality of life total score (PedsQL) <70 (28), Sleep disturbance (ISI) ≥9 (29, 30), and School absence ≥20% (27). The cut-off values were determined *a priori*, primarily based on the previous literature outlined in Section 2.4.1, which defines the HICP variables. All children had data for at least 3 items and therefore none were excluded due to missing data.

Logistic regression models were constructed to investigate potential characteristics and clinical factors in children and parents that were associated with HICP. Univariable models were constructed for the 17 potential associated variables. All associated variables with a *p*-value lower than 0.3 were then included in the final multivariate models. Since many variables were significant, one multivariable model was made using the variables that were associated with the child, and one model was made using variables that were associated with the parent(s). A two-tailed significance level of *p* = 0.05 defined significance.

A formal power analysis was not conducted because this was a registry-based study with a fixed sample size determined by the number of eligible cases during the study period. This is in accordance with the STROBE guidelines.

Statistical analysis was performed in R version 4.3.3 (reference: R Core Team (2024). R: A language and environment for statistical computing. R Foundation for Statistical Computing, Vienna, Austria. URL https://www.R-project.org/.)

## 3 Results

### 3.1 Participants

In Table 1 descriptive statistics for the 484 children and parents are presented. A total of 289 (59.7%) of the children were considered to have HICP according to the definition used here. Overall, missing data were minimal, as all information was collected during the initial clinic visit and any incomplete questionnaire responses were completed upon arrival.

**Table 1:** Descriptive statistics for children with chronic pain and their parents divided into groups of low and high impact chronic pain.

Variables	Low impact chronic pain *N* = 195^a^	High impact chronic pain *N* = 289^a^	Total *N* = 484^a^
*Variables related to the child*			
Age			
Mean (SD)	14.0 (2.17)	14.5 (2.13)	14.3 (2.16)
Median [Min, Max]	14.0 [10.0, 18.0]	15.0 [10.0, 18.0]	15.0 [10.0, 18.0]
Sex			
Female	135 (69.2%)	214 (74.0%)	349 (72.1%)
Male	60 (30.8%)	75 (26.0%)	135 (27.9%)
Months since pain started			
Mean (SD)	47.5 (39.4)	50.4 (39.9)	50.4 (39.9)
Median [Min, Max]	36.0 [1.50, 204]	38.5 [3.00, 190]	38.5 [3.00, 190]
Missing	0 (0%)	1 (0.3%)	1 (0.3%)
Pain location			
Headache	63 (32,3%)	77 (26.6%)	140 (28.9%)
Abdominal pain	38 (19.5%)	59 (20.4%)	97 (20.0%)
Back or neck pain	23 (11.8%)	34 (11.8%)	57 (11.8%)
Musculoskeletal pain	56 (28.7%)	62 (21.5%)	118 (24.4%)
Widespread pain	15 (7.7%)	57 (19.7%)	72 (14.9%)
Number of pain locations			
One	80 (41.0%)	82 (28.4%)	162 (33.5%)
Two	30 (15.4%)	30 (10.4%)	60 (12.4%)
Three or more	85 (43.6%)	177 (61.2%)	262 (54.1%)
Depression			
Mean (SD)	16.2 (8.55)	28.4 (11.1)	23.5 (11.8)
Median [Min, Max]	15.0 [0, 46.0]	28.0 [3.00, 58.0]	22.0 [0, 58.0]
Missing	4 (2.1%)	4 (1.4%)	8 (1.7%)
Psychological Inflexibility			
Mean (SD)	42.4 (12.4)	57.4 (13.6)	51.3 (15.0)
Median [Min, Max]	41.0 [15.0, 80.0]	58.0 [15.0, 84.0]	51.0 [15.0, 84.0]
Missing	1 (0.5%)	5 (1.7%)	6 (1.2%)
Pain reactivity			
Mean (SD)	12.8 (6.94)	18.5 (7.26)	16.2 (7.67)
Median [Min, Max]	13.0 [0, 30.0]	19.0 [0, 30.0]	16.0 [0, 30.0]
Missing	0 (0%)	1 (0.3%)	1 (0.2%)
*Variables related to the reporting parent*			
Age			
Mean (SD)	45.9 (4.93)	45.5 (5.87)	45.7 (5.50)
Median [Min, Max]	46.0 [32.0, 59.0]	46.0 [30.0, 72.0]	46.0 [30.0, 72.0]
Missing	1 (0.5%)	6 (2.1%)	7 (1.4%)
Sex			
Female	157 (80.5%)	230 (79.6%)	387 (80.0%)
Male	37 (19.0%)	54 (18.7%)	91 (18.8%)
Missing	1 (0.5%)	5 (1.7%)	6 (1.2%)
Highest education			
High school or less	68 (34.9%)	143 (49.5%)	211 (43.6%)
College or university	125 (64.1%)	139 (48.1%)	264 (54.5%)
Missing	2 (1.0%)	7 (2.4%)	9 (1.9%)
Marital status			
Married + partnership	165 (84.6%)	223 (77.2%)	388 (80.2%)
Single/widowed	29 (14.9%)	61 (21.1%)	90 (18.6%)
Missing	1 (0.5%)	5 (1.7%)	6 (1.2%)
Employment			
Employed	169 (86.7%)	221 (76.5%)	390 (80.6%)
Not employed	25 (12.8%)	63 (21.8%)	88 (18.2%)
Missing	1 (0.5%)	5 (1.7%)	6 (1.2%)
Chronic pain, both parents			
No	66 (33.8%)	84 (29.1%)	150 (31.0%)
Yes—one parent	75 (38.5%)	105 (36.3%)	180 (37.2%)
Yes—both parents	34 (17.4%)	76 (26.3%)	110 (22.7%)
Missing	20 (10.3%)	24 (8.3%)	44 (9.1%)
Psychological flexibility			
Mean (SD)	32.1 (10.3)	26.7 (11.6)	28.9 (11.4)
Median [Min, Max]	32.5 [5.00, 52.0]	26.0 [0, 55.0]	29.0 [0, 55.0]
Missing	13 (6.7%)	23 (8.0%)	36 (7.4%)
Pain reactivity			
Mean (SD)	16.6 (7.22)	20.3 (7.17)	18.8 (7.41)
Median [Min, Max]	17.0 [0, 30.0]	21.0 [1.00, 30.0]	19.0 [0, 30.0]
Missing	3 (1.5%)	3 (1.0%)	(1.2%)

a *n* (%).

There were more girls than boys among the participants (72.1% vs. 27.9%), but the distribution for the respective sex in the LICP and HICP groups was comparable. Mean age was around 14 years in both groups. Headache was the most common pain location (28.9%), followed by musculoskeletal pain (24.4%) and abdominal pain (20.0%) in the overall study group, with the same ranking observed in both comparison groups. Widespread pain was more common in the high impact group (19.7%) than in the low (7.7%). More than half of the participants had pain in ≥3 locations, but a higher percentage of multiple pain locations was found in the high impact group (61.2%) compared to the low impact group (43.6%).

Most accompanying parents were mothers 387 (80.0%), who completed the questionnaires. The percentage of mothers and fathers attending were equal in the high and low impact groups. The level of parental education was higher in the low impact group, (64.1%) compared to the high impact group (48.1%). Parents were generally employed to a high degree (80.6%), but there were more employed parents in the low-impact group than in the high impact group. Most parents were married or in a partnership (80.2%), with a slight predominance of single parents (21.1%) in the group with high impact on child functioning compared to (14.9%) in the low impact group. Sixty percent of parents reported experiencing chronic pain themselves. Families in which both parents had chronic pain were more common in the high impact group (26.3%) compared to the low impact group (17.4%). However, there was no difference between the groups when only one parent had chronic pain.

### 3.2 Multivariable logistic regression analysis

The multivariable logistic regression analyses are described in detail in Tables 2, 3. A multitude of child variables (age, primary pain location, number of pain locations, symptoms of depression, psychological inflexibility and pain reactivity) and parental variables (higher educational level, psychological flexibility and pain reactivity) had a *p*-value lower than 0.3 and were included in the final multivariate models. Variables associated with children were analyzed separately from variables associated with parents. Only a few variables were significantly associated with HICP in the multivariate models. Among the child variables symptoms of depression and psychological inflexibility were significant for HICP. When analyzing variables associated with parents, pain reactivity was significantly associated with HICP.

**Table 2:** Results for the multivariable logistic regression analysis focused on child variables associated with high impact chronic pain in children (10–18 years) in a specialized pediatric pain clinic in Sweden.

Variables	Odds ratios (95% CI)	*p*-value
Age	1.075 (0.96; 1.205)	0.212
Sex - male	1.134 (0.66; 1.961)	0.649
Pain location (reference category: headache)		0.465
Abdominal	1.175 (0.596; 2.324)	
Back or neck	1.213 (0.557; 2.664)	
Musculoskeletal	0.867 (0.459; 1.636)	
WSP	1.949 (0.808; 4.853)	
Number of pain locations (reference category: one)		0.540
Two	0.675 (0.317; 1.423)	
Three or more	0.799 (0.45; 1.408)	
Depression	1.101 (1.07; 1.134)	<0.0001
Psychological inflexibility	1.064 (1.043; 1.088)	<0.0001
Pain reactivity	0.99 (0.95; 1.031)	0.616

*N* = 484, α = 0.05.

CI, confidence interval; WSP, widespread pain.

**Table 3:** Results for the multivariable logistic regression analysis focused on parental variables associated with high impact chronic pain in children (10–18 years) in a specialized pediatric pain clinic in Sweden.

Variables	Odds ratios (95% CI)	*p*-value
Parental education (college or university)	0.782 (0.501; 1.219)	0.277
Marital status (living apart relationship, single, or widowed)	1.34 (0.756; 2.424)	0.322
Employment rate (not employed)	1.542 (0.83; 2.948)	0.178
Parental pain (reference category: no)		0.267
Yes—one parent	1.09 (0.672; 1.769)	
Yes—both parents	1.571 (0.892; 2.795)	
Parental psychological flexibility	0.98 (0.953; 1.007)	0.143
Parental pain reactivity	1.05 (1.009; 1.094)	0.018

*N* = 484, α = 0.05.

CI, confidence interval.

## 4 Discussion

The aims of the present exploratory study were to define HICP from a wider biopsychosocial perspective and to investigate characteristics and clinical factors in children and parents as variables associated with HICP, as indicated by pain severity, pain interference, overall well-being, sleep disturbance and school absence. The major findings were that symptoms of depression and psychological inflexibility in children, as well as pain reactivity in parents were associated with HICP. Several sociodemographic and pain-related factors in children and parents differed between the groups of children with high and low impact chronic pain.

This study adds to the very few studies investigating HICP in children (4, 17, 18). Previous studies have used a limited and narrower definition of HICP, leaving out psychosocial aspects of the biopsychosocial model in their classification attempts (4, 5, 17–20). As a result, these approaches do not fully reflect the biopsychosocial principles emphasized in current international pediatric chronic pain guidelines (3, 55). Neither parental nor behavioral aspects have been included, making the present study unique, as both variables have been described as important factors for the development of chronic pain (56). Our proposed definition builds upon previous conceptualizations of HICP, integrating all components of the biopsychosocial model and encompassing the recommended mandatory and key outcome domains for pediatric chronic pain research that are relevant to baseline assessment (24). In doing so, we align with existing international guidelines while further refining the assessment of high impact chronic pain in children. This approach resonates with the biopsychosocial principles emphasized in contemporary frameworks such as the WHO guidelines, the Lancet Child & Adolescent Health Commission, the European Pain Federation (EFIC) pain recommendations, and the IASP/ Special Interest Group (SIG) on pediatric pain statements (3, 55, 57, 58). By incorporating these perspectives, our definition not only reflects current best practice but also advances the conceptualization of HICP in pediatric populations.

Children with complex symptoms should be managed in tertiary care, a recommendation supported by our findings, as the majority (59.7%) were classified as having HICP. A similar proportion (63%) was reported in a German tertiary care study (18), which contrasts sharply with school-based studies where only 5%–10% of children were identified as having HICP, likely due to differences in population severity and assessment context. School samples represent the general population with mostly mild pain, whereas tertiary care should include the moste severe and complex cases (4, 17).

All participants had experienced pain for more than three months, consistent with the International Association for the Study of Pain (IASP) definition of chronic pain. It could be hypothesized that a longer duration of pain would be associated with greater impairment in daily functioning. However, our analyses revealed no apperent difference between the HICP and LICP groups with respect to the mean duration of pain prior to assessment. These findings suggest that pain duration alone is unlikely to account for the greater impact on daily life observed among children with high impact chronic pain.

When investigating child-related variables associated with HICP, only higher levels of depressive symptoms and psychological inflexibility turned out significant. Depression is together with anxiety the most common psychiatric comorbidity in children with chronic pain (59–61) with a prevalence which is more than three times higher than in the general population (62). Previous studies have shown relations between psychiatric comorbidity and increased pain, poorer quality of life, and reduced functioning in everyday life (63–67), as well as an increased risk of continued problems into adulthood (14). This underscores the critical role of depressive symptoms in HICP and highlights the necessity of addressing mental health within pediatric chronic pain treatment, particularly in tertiary care settings (3, 62, 64, 68). One treatment strategy with proven effect for depression is Cognitive behavioral therapy (CBT). Since this treatment paradigm is the most used within chronic pain treatment, CBT strategies to specifically target depression could easily be integrated in IPR (69) or possibly used outside specialized pain care to prevent HICP (68).

As mentioned earlier, higher baseline values of psychological inflexibility (consisting of items assessing pain avoidance and cognitive fusion) were associated with HICP in this study. This finding is important for the choice of interventions in the rehabilitation of children with chronic pain. Psychological flexibility is about acting in line with one's chosen values in the presence of unwanted thoughts, emotions or bodily symptoms. While psychological flexibility promotes resilience and action, psychological inflexibility captures opposite processes such as behavioral avoidance, restriction and inactivity (51). Acceptance and Commitment Therapy (ACT) is a treatment focusing on wellbeing and valued activity through heightened psychological flexibility (70). The findings in the present study are in agreement with earlier studies in adults with chronic pain, where psychological inflexibility has been shown to predict worse treatment outcomes (71, 72). Specific screening and intensive targeting of psychological inflexibility using treatment strategies from ACT during treatment may be beneficial for individuals with HICP.

Psychological flexibility in parents, as assessed by PPFQ, has repeatedly been found to be negatively correlated to child functioning in earlier studies (52, 54), but no significant difference was found in the present study when comparing groups of high and low impact chronic pain. This in contrast to the finding regarding psychological inflexibility in children.

When examining parent-related variables associated with HICP, only pain reactivity, specifically parental worry about the child's pain, was significant. Such parental responses may influence the child's psychological flexibility, potentially reinforcing maladaptive coping pattern (54). This finding is also consistent with the fear-avoidance model, a CBT-based framework for chronic pain with strong empirical support. The model emphasizes pain-related worry, captured in this study, as well as catastrophizing and avoidance behaviors, which have been extensively documented in adults (51, 73). This model is also applicable for children, but in such instances, it needs to be contextual and reflect the interaction between children and parents (74, 75). Parents' reactions such as catastrophizing, worrying and protective behavior with regard to the child's pain have been shown to have a negative impact on the child's reactions and to trigger avoidance behaviors in relation to pain (75–77). The findings of this study underscore the importance of interventions that address parental responses, such as pain reactivity, in the rehabilitation of children with chronic pain.

The strengths of this study include its large sample size (484 participants), the breadth of variables examined, and the inclusion of parental factors in a real-world clinical setting, as all children aged 10 years or older admitted for assessment at the SPPC were included. However, the findings should be interpreted in light of certain limitations. In accordance with the STROBE guidelines, no significance tests were performed to describe differences in characteristics between the HICP and LICP groups in Table 1.

Another limitation is the retrospective design and registry data which obviously only allow for cross-sectional and no causality analyses. While we can anticipate that the associated factors, such as symptoms of depression, greater psychological inflexibility, and/or parental pain reactivity, may contribute to an escalation in pain impact, they could equally be consequences of high pain-related interference in daily life. Although we believe these factors play a role in the maintenance of pain, this conclusion cannot be drawn from the present data. Future research is needed to investigate these relationships further.

The cut-off values used to define HICP in this study were primarily informed by prior literature. However, we acknowledge that a more robust justification is warranted. In this exploratory study, no sensitivity analyses were conducted to test alternative thresholds. Further studies should focus on validating the selected variables and their corresponding cut-offs to strengthen reliability and generalizability.

The participants were all attending a specialized pediatric pain clinic, and the results cannot be generalized to children with chronic pain in other clinical contexts. As in most studies, the majority of parents accompanying their child to the clinic were mothers, which may have influenced findings related to parental factors and underscores the importance of including fathers in future research. Self-report data from questionnaires were used to identify symptoms of depression which is not sufficient as a diagnostic tool and self-report scales can only provide a provisional diagnosis.

The suggested core outcome set of measures for pediatric pain studies was followed as far as possible (24). However, because not all recommended questionnaires are translated and validated for children in Swedish, a few non-validated questionnaires were used, e.g., the PRS. However, it is commonly used and has been proven sufficient in capturing pain-related reactions (46, 78). The PIPS, which was used to assess psychological inflexibility in the participating children, has demonstrated acceptable validity and reliability in children and adults (47–50). Still, the representativity of the total score as a measure of all aspects of psychological (in)flexibility has been questioned in some studies (48, 79). Another limitation is the risk of bias, since the pain physician involved in the assessment was also the first author of the present study.

Given the high costs and limited availability of IPR, it is crucial to further investigate which children truly benefit from this intervention. Additionally, the subgroup classified as having LICP warrants closer examination to better understand their needs. This group may include at least two distinct subcategories: one that could benefit from support within primary care, and another that may require only clear explanations, reassurance, and self-management strategies. Gaining a better understanding could help optimize the use of resources and ensure that children are directed to the most appropriate care pathways.

In conclusion, further development of the definition of HICP is proposed using a broader biopsychosocial perspective to more fully capture the complexity of these symptoms. This study also highlight symptoms of depression and psychological inflexibility in children, as well as parental pain reactivity, as associated with HICP. These findings underscore the importance of taking psychiatric comorbidity and child and parental behavioral aspects into account when tailoring interventions for children with chronic pain. Specific assessment and targeting of depressive symptoms and psychological inflexibility in children, and pain reactivity in parents, during pain rehabilitation may improve treatment results in terms of functioning and reduce the risk of ongoing challenges into adulthood, especially in individuals with HICP.

## Data Availability

The datasets presented in this article are not readily available because the data that support the findings of this study are not openly available due to reasons of sensitivity. Requests to access the datasets should be directed to ulla.caverius@med.lu.se.

## References

[1] Chambers CT Dol J Tutelman PR Langley CL Parker JA Cormier BT The prevalence of chronic pain in children and adolescents: a systematic review update and meta-analysis. Pain. (2024) 165(10):2215–34. 10.1097/j.pain.0000000000003267 38743558 PMC11404345

[2] King S Chambers CT Huguet A MacNevin RC McGrath PJ Parker L The epidemiology of chronic pain in children and adolescents revisited: a systematic review. Pain. (2011) 152(12):2729–38. 10.1016/j.pain.2011.07.016 22078064

[3] WHO. Guidelines on the Management of Chronic Pain in Children. Geneva: World Health Organization (2020). Available online at: https://www.who.int/publications/i/item/9789240017870 (Accessed November 12, 2025). 33433967

[4] Miro J Roman-Juan J Sanchez-Rodriguez E Sole E Castarlenas E Jensen MP. Chronic pain and high impact chronic pain in children and adolescents: a cross-sectional study. J Pain. (2023) 24(5):812–23. 10.1016/j.jpain.2022.12.007 36577459

[5] Pitcher MH Von Korff M Bushnell MC Porter L. Prevalence and profile of high-impact chronic pain in the United States. J Pain. (2019) 20(2):146–60. 10.1016/j.jpain.2018.07.006 30096445 PMC8822465

[6] Gatchel RJ Peng YB Peters ML Fuchs PN Turk DC. The biopsychosocial approach to chronic pain: scientific advances and future directions. Psychol Bull. (2007) 133(4):581–624. 10.1037/0033-2909.133.4.581 17592957

[7] Liossi C Howard RF. Pediatric chronic pain: biopsychosocial assessment and formulation. Pediatrics. (2016) 138(5):e20160331. 10.1542/peds.2016-0331 27940762

[8] Alfven G Caverius U Nilsson SR. Pain in children and adolescents a neglected area: deficiencies in care according to a questionnaire. Lakartidningen. (2012) 109(19):966–7. 22734263

[9] Gåve E Berg K Caverius U Ekström G Grinsvall C Lanzinger M Nationellt vårdprogram för långvarig smärta hos barn. Nationellt system för kunskapsstyrning hälso- och sjukvård, Sveriges regioner i samverkan (2023). Available online at: https://vardpersonal.1177.se/globalassets/nkk/nationell/media/dokument/kunskapsstod/vardprogram/nationellt-vardprogram-for-langvarig-smarta-hos-barn.pdf (Accessed February 02, 2025). 39354734

[10] Brattberg G. Do pain problems in young school children persist into early adulthood? A 13-year follow-up. Eur J Pain. (2004) 8(3):187–99. 10.1016/j.ejpain.2003.08.001 15109969

[11] Walker LS Sherman AL Bruehl S Garber J Smith CA. Functional abdominal pain patient subtypes in childhood predict functional gastrointestinal disorders with chronic pain and psychiatric comorbidities in adolescence and adulthood. Pain. (2012) 153(9):1798–806. 10.1016/j.pain.2012.03.026 22721910 PMC3413740

[12] Hassett AL Hilliard PE Goesling J Clauw DJ Harte SE Brummett CM. Reports of chronic pain in childhood and adolescence among patients at a tertiary care pain clinic. J Pain. (2013) 14(11):1390–7. 10.1016/j.jpain.2013.06.010 24021576

[13] Stone AL Epstein I Bruehl S Garber J Smith CA Walker LS. Twenty-year outcomes of a pediatric chronic abdominal pain cohort: early adulthood health Status and offspring physical and behavioral health. J Pain. (2023) 24(1):145–56. 10.1016/j.jpain.2022.09.007 36126817 PMC9789180

[14] Murray CB Groenewald CB de la Vega R Palermo TM. Long-term impact of adolescent chronic pain on young adult educational, vocational, and social outcomes. Pain. (2020) 161(2):439–45. 10.1097/j.pain.0000000000001732 31651579 PMC7001863

[15] Murray CB Li R Kashikar-Zuck S Zhou C Palermo TM. Adolescent predictors of young adult pain and health outcomes: results from a 6-year prospective follow-up study. Pain. (2025) 166(1):42–51. 10.1097/j.pain.0000000000003308 38916525 PMC12169839

[16] Wager J Ruhe AK Stahlschmidt L Leitsch K Claus BB Hauser W Long-term outcomes of children with severe chronic pain: comparison of former patients with a community sample. Eur J Pain. (2021) 25(6):1329–41. 10.1002/ejp.1754 33619774

[17] Huguet A Miro J. The severity of chronic pediatric pain: an epidemiological study. J Pain. (2008) 9(3):226–36. 10.1016/j.jpain.2007.10.015 18088558

[18] Wager J Hechler T Darlington AS Hirschfeld G Vocks S Zernikow B. Classifying the severity of paediatric chronic pain - an application of the chronic pain grading. Eur J Pain. (2013) 17(9):1393–402. 10.1002/j.1532-2149.2013.00314.x 23576527

[19] Von Korff M DeBar LL Krebs EE Kerns RD Deyo RA Keefe FJ. Graded chronic pain scale revised: mild, bothersome, and high-impact chronic pain. Pain. (2020) 161(3):651–61. 10.1097/j.pain.0000000000001758 31764390 PMC7097879

[20] George SZ Allen KD Alvarez C Arbeeva L Callahan LF Nelson AE Prevalence and factors associated with high-impact chronic pain in knee osteoarthritis: the johnston county health study. J Pain. (2024) 25(12):104687. 10.1016/j.jpain.2024.104687 39343191 PMC12240002

[21] Fisher E Law E Dudeney J Palermo TM Stewart G Eccleston C. Psychological therapies for the management of chronic and recurrent pain in children and adolescents. Cochrane Database Syst Rev. (2018) 9:CD003968. 10.1002/14651858.CD003968.pub5 30270423 PMC6257251

[22] Wager J Szybalski K Schenk S Frosch M Zernikow B. Predictors of treatment outcome in children with medically unexplained pain seeking primary care: a prospective cohort study. Eur J Pain. (2019) 23:1507–18. 10.1002/ejp.1426 31112345

[23] McGrath PJ Walco GA Turk DC Dworkin RH Brown MT Davidson K Core outcome domains and measures for pediatric acute and chronic/recurrent pain clinical trials: PedIMMPACT recommendations. J Pain. (2008) 9(9):771–83. 10.1016/j.jpain.2008.04.007 18562251

[24] Palermo TM Walco GA Paladhi UR Birnie KA Crombez G de la Vega R Core outcome set for pediatric chronic pain clinical trials: results from a Delphi poll and consensus meeting. Pain. (2021) 162(10):2539–47. 10.1097/j.pain.0000000000002241 33625074 PMC8442740

[25] Caverius U Åkerblom S Lexell J Fischer MR. Characteristics of children with persistent pain and their parents in a tertiary interdisciplinary pain clinic. Paediatr Neonatal Pain. (2025) 7(2):e70005. 10.1002/pne2.70005 40226841 PMC11992596

[26] von Elm E Altman DG Egger M Pocock SJ Gotzsche PC Vandenbroucke JP The strengthening the reporting of observational studies in epidemiology (STROBE) statement: guidelines for reporting observational studies. Lancet. (2007) 370(9596):1453–7. 10.1016/S0140-6736(07)61602-X 18064739

[27] Zernikow B Wager J Hechler T Hasan C Rohr U Dobe M Characteristics of highly impaired children with severe chronic pain: a 5-year retrospective study on 2249 pediatric pain patients. BMC Pediatr. (2012) 12:54. 10.1186/1471-2431-12-54 22591492 PMC3404028

[28] Huang IC Thompson LA Chi YY Knapp CA Revicki DA Seid M The linkage between pediatric quality of life and health conditions: establishing clinically meaningful cutoff scores for the PedsQL. Value Health. (2009) 12(5):773–81. 10.1111/j.1524-4733.2008.00487.x 19508660 PMC4299816

[29] Chung KF Kan KK Yeung WF. Assessing insomnia in adolescents: comparison of insomnia severity index, Athens insomnia scale and sleep quality index. Sleep Med. (2011) 12(5):463–70. 10.1016/j.sleep.2010.09.019 21493134

[30] Kanstrup M Holmstrom L Ringstrom R Wicksell RK. Insomnia in paediatric chronic pain and its impact on depression and functional disability. Eur J Pain. (2014) 18(8):1094–102. 10.1002/j.1532-2149.2013.00450.x 24443275

[31] Logan DE Simons LE Stein MJ Chastain L. School impairment in adolescents with chronic pain. J Pain. (2008) 9(5):407–16. 10.1016/j.jpain.2007.12.003 18255341

[32] Groenewald CB Giles M Palermo TM. School absence associated with childhood pain in the United States. Clin J Pain. (2019) 35(6):525–31. 10.1097/AJP.0000000000000701 30844952 PMC6502652

[33] Birnie KA Hundert AS Lalloo C Nguyen C Stinson JN. Recommendations for selection of self-report pain intensity measures in children and adolescents: a systematic review and quality assessment of measurement properties. Pain. (2019) 160(1):5–18. 10.1097/j.pain.0000000000001377 30180088

[34] von Baeyer CL Spagrud LJ McCormick JC Choo E Neville K Connelly MA. Three new datasets supporting use of the numerical rating scale (NRS-11) for children's self-reports of pain intensity. Pain. (2009) 143(3):223–7. 10.1016/j.pain.2009.03.002 19359097

[35] Holmstrom L Kemani MK Kanstrup M Wicksell RK. Evaluating the statistical properties of the pain interference index in children and adolescents with chronic pain. J Dev Behav Pediatr. (2015) 36(6):450–4. 10.1097/DBP.0000000000000191 26154714

[36] Kemani MK Zetterqvist V Kanstrup M Holmstrom L Wicksell RK. A validation of the pain interference index in adults with long-standing pain. Acta Anaesthesiol Scand. (2016) 60(2):250–8. 10.1111/aas.12599 26310686

[37] Wicksell RK Melin L Lekander M Olsson GL. Evaluating the effectiveness of exposure and acceptance strategies to improve functioning and quality of life in longstanding pediatric pain–a randomized controlled trial. Pain. (2009) 141(3):248–57. 10.1016/j.pain.2008.11.006 19108951

[38] Varni JW Limbers CA Burwinkle TM. How young can children reliably and validly self-report their health-related quality of life?: an analysis of 8,591 children across age subgroups with the PedsQL 4.0 generic core scales. Health Qual Life Outcomes. (2007) 5:1. 10.1186/1477-7525-5-1 17201920 PMC1769360

[39] Petersen S Hagglof B Stenlund H Bergstrom E. Psychometric properties of the Swedish PedsQL, pediatric quality of life inventory 4.0 generic core scales. Acta Paediatr. (2009) 98(9):1504–12. 10.1111/j.1651-2227.2009.01360.x 19570060

[40] Bastien CH Vallieres A Morin CM. Validation of the insomnia severity index as an outcome measure for insomnia research. Sleep Med. (2001) 2(4):297–307. 10.1016/S1389-9457(00)00065-4 11438246

[41] Morin CM Belleville G Belanger L Ivers H. The insomnia severity index: psychometric indicators to detect insomnia cases and evaluate treatment response. Sleep. (2011) 34(5):601–8. 10.1093/sleep/34.5.601 21532953 PMC3079939

[42] von Baeyer CL Lin V Seidman LC Tsao JC Zeltzer LK. Pain charts (body maps or manikins) in assessment of the location of pediatric pain. Pain Manag. (2011) 1(1):61–8. 10.2217/pmt.10.2 21572558 PMC3091382

[43] Fendrich M Weissman MM Warner V. Screening for depressive disorder in children and adolescents: validating the center for epidemiologic studies depression scale for children. Am J Epidemiol. (1990) 131(3):538–51. 10.1093/oxfordjournals.aje.a115529 2301363

[44] Olsson G von Knorring AL. Depression among Swedish adolescents measured by the self-rating scale center for epidemiology studies-depression child (CES-DC). Eur Child Adolesc Psychiatry. (1997) 6(2):81–7. 10.1007/s007870050012 9257089

[45] Wicksell RK Olsson GL Hayes SC. Mediators of change in acceptance and commitment therapy for pediatric chronic pain. Pain. (2011) 152(12):2792–801. 10.1016/j.pain.2011.09.003 21995881

[46] Kanstrup M Wicksell RK Kemani M Wiwe Lipsker C Lekander M Holmstrom L. A clinical pilot study of individual and group treatment for adolescents with chronic pain and their parents: effects of acceptance and commitment therapy on functioning. Children. (2016) 3(4):30. 10.3390/children3040030 27854323 PMC5184805

[47] Wicksell RK Lekander M Sorjonen K Olsson GL. The psychological inflexibility in pain scale (PIPS)–statistical properties and model fit of an instrument to assess change processes in pain related disability. Eur J Pain. (2010) 14(7):771 e1–14. 10.1016/j.ejpain.2009.11.015 20106685

[48] Hester R Trompetter EB van Baalen B Klein M Köke A Reneman MF The psychological inflexibility in pain scale (PIPS). Eur J Psychol Assess. (2014) 30(4):289–95. 10.1027/1015-5759/a000191

[49] Nagasawa Y Shibata A Fukamachi H Ishii K Wicksell RK Oka K. The psychological inflexibility in pain scale (PIPS): validity and reliability of the Japanese version for chronic low back pain and knee pain. J Pain Res. (2021) 14:325–32. 10.2147/JPR.S287549 33568939 PMC7870289

[50] Ghomian S Naseri M Masumian S Zadeh T Nuri N. Psychometric feature of the child and parent versions of psychological inflexibility in pain scale (PIPS) in children with chronic pain and their parents. Caspian J Pediatr. (2017) 3(2):241–7. 10.22088/acadpub.BUMS.3.2.241

[51] McCracken LM Morley S. The psychological flexibility model: a basis for integration and progress in psychological approaches to chronic pain management. J Pain. (2014) 15(3):221–34. 10.1016/j.jpain.2013.10.014 24581630

[52] Timmers I Simons LE Hernandez JM McCracken LM Wallace DP. Parent psychological flexibility in the context of pediatric pain: brief assessment and associations with parent behaviour and child functioning. Eur J Pain. (2019) 23(7):1340–50. 10.1002/ejp.1403 31002473

[53] Wiwe Lipsker C Kanstrup M Holmstrom L Kemani M Wicksell RK. The parent psychological flexibility questionnaire (PPFQ): item reduction and validation in a clinical sample of Swedish parents of children with chronic pain. Children. (2016) 3(4):32. 10.3390/children3040032 27869780 PMC5184807

[54] McCracken LM Gauntlett-Gilbert J. Role of psychological flexibility in parents of adolescents with chronic pain: development of a measure and preliminary correlation analyses. Pain. (2011) 152(4):780–5. 10.1016/j.pain.2010.12.001 21292397

[55] Eccleston C Fisher E Howard RF Slater R Forgeron P Palermo TM Delivering transformative action in paediatric pain: a lancet child & adolescent health commission. Lancet Child Adolesc Health. (2021) 5(1):47–87. 10.1016/S2352-4642(20)30277-7 33064998

[56] McKillop HN Banez GA. A broad consideration of risk factors in pediatric chronic pain: where to go from here? Children. (2016) 3(4):38. 10.3390/children3040038 27916884 PMC5184813

[57] Finley G Franck L Grunau R vonBaeyer C. Pain: clinical updates: why children’s pain matters. Int Assoc Study Pain. (2005) 13.

[58] EFIC. Pain in children: management [fact sheet]. European Pain Federation (EFIC) (2019). Available online at: https://europeanpainfederation.eu/wp-content/uploads/2019/02/8-Pain-in-Children-Management555.pdf (Accessed December 22, 2025).

[59] Eccleston C Crombez G Scotford A Clinch J Connell H. Adolescent chronic pain: patterns and predictors of emotional distress in adolescents with chronic pain and their parents. Pain. (2004) 108(3):221–9. 10.1016/j.pain.2003.11.008 15030941

[60] Vinall J Pavlova M Asmundson GJ Rasic N Noel M. Mental health comorbidities in pediatric chronic pain: a narrative review of epidemiology, models, neurobiological mechanisms and treatment. Children. (2016) 3(4):40. 10.3390/children3040040 27918444 PMC5184815

[61] Arts J Gubbels JS Verhoeff AP Chinapaw MJM Lettink A Altenburg TM. A systematic review of proxy-report questionnaires assessing physical activity, sedentary behavior and/or sleep in young children (aged 0–5 years). Int J Behav Nutr Phys Act. (2022) 19(1):18. 10.1186/s12966-022-01251-x 35164783 PMC8845346

[62] Dudeney J Aaron RV Hathway T Bhattiprolu K Bisby MA McGill LS Anxiety and depression in youth with chronic pain: a systematic review and meta-analysis. JAMA Pediatr. (2024) 178(11):1114–23. 10.1001/jamapediatrics.2024.3039 39250143 PMC11385330

[63] Gauntlett-Gilbert J Eccleston C. Disability in adolescents with chronic pain: patterns and predictors across different domains of functioning. Pain. (2007) 131(1–2):132–41. 10.1016/j.pain.2006.12.021 17267129

[64] Simons LE Sieberg CB Claar RL. Anxiety and impairment in a large sample of children and adolescents with chronic pain. Pain Res Manag. (2012) 17(2):93–7. 10.1155/2012/420676 22518371 PMC3393050

[65] Vetter TR McGwin G Jr. Bridgewater CL Madan-Swain A Ascherman LI. Validation and clinical application of a biopsychosocial model of pain intensity and functional disability in patients with a pediatric chronic pain condition referred to a subspecialty clinic. Pain Res Treat. (2013) 2013:143292. 10.1155/2013/143292 24251035 PMC3819919

[66] Cunningham NR Cohen MB Farrell MK Mezoff AG Lynch-Jordan A Kashikar-Zuck S. Concordant parent-child reports of anxiety predict impairment in youth with functional abdominal pain. J Pediatr Gastroenterol Nutr. (2015) 60(3):312–7. 10.1097/MPG.0000000000000625 25714575 PMC4341941

[67] Soltani S Kopala-Sibley DC Noel M. The co-occurrence of pediatric chronic pain and depression: a narrative review and conceptualization of mutual maintenance. Clin J Pain. (2019) 35(7):633–43. 10.1097/AJP.0000000000000723 31094934

[68] Tegethoff M Belardi A Stalujanis E Meinlschmidt G. Comorbidity of mental disorders and chronic pain: chronology of onset in adolescents of a national representative cohort. J Pain. (2015) 16(10):1054–64. 10.1016/j.jpain.2015.06.009 26168877

[69] NICE Evidence Reviews Collection. Psychological Interventions for the Treatment of Depression: Depression in Children and Young People, 2019 Evidence Review: Evidence Review A. London: National Institute for Health and Care Excellence (NICE). Copyright © NICE 2019 (2019). 32479041

[70] Hayes SC Strosahl KD Wilson KG. Acceptance and Commitment Therapy: An Experiential Approach to Behavior Change. New York: Guilford Press. (1999).

[71] Akerblom S Perrin S Rivano Fischer M McCracken LM. Predictors and mediators of outcome in cognitive behavioral therapy for chronic pain: the contributions of psychological flexibility. J Behav Med. (2021) 44(1):111–22. 10.1007/s10865-020-00168-9 32642875 PMC7846536

[72] Gilpin HR Stahl DR McCracken LM. A theoretically guided approach to identifying predictors of treatment outcome in contextual cognitive behavioural therapy for chronic pain. Eur J Pain. (2019) 23(2):354–66. 10.1002/ejp.1310 30176099

[73] Vlaeyen JW Linton SJ. Fear-avoidance and its consequences in chronic musculoskeletal pain: a state of the art. Pain. (2000) 85(3):317–32. 10.1016/S0304-3959(99)00242-0 10781906

[74] Simons LE Kaczynski KJ. The fear avoidance model of chronic pain: examination for pediatric application. J Pain. (2012) 13(9):827–35. 10.1016/j.jpain.2012.05.002 22832693 PMC3438388

[75] Goubert L Simons LE. Cognitive styles and proceses in paediatric pain. In: McGrath PJ Stevens BJ Walker SM, editors. Oxford Textbook of Paediatric Pain. Oxford: Oxford University Press (2013). p. 95–101.

[76] Chow ET Otis JD Simons LE. The longitudinal impact of parent distress and behavior on functional outcomes among youth with chronic pain. J Pain. (2016) 17(6):729–38. 10.1016/j.jpain.2016.02.014 26993960 PMC4885759

[77] Simons LE Smith A Kaczynski K Basch M. Living in fear of your child’s pain: the parent fear of pain questionnaire. Pain. (2015) 156(4):694–702. 10.1097/j.pain.0000000000000100 25630026 PMC4366345

[78] Wicksell RK Kanstrup M Kemani MK Holmstrom L. Pain interference mediates the relationship between pain and functioning in pediatric chronic pain. Front Psychol. (2016) 7:1978. 10.3389/fpsyg.2016.01978 28082931 PMC5183613

[79] Barke A Riecke J Rief W Glombiewski JA. The psychological inflexibility in pain scale (PIPS) - validation, factor structure and comparison to the chronic pain acceptance questionnaire (CPAQ) and other validated measures in German chronic back pain patients. BMC Musculoskelet Disord. (2015) 16:171. 10.1186/s12891-015-0641-z 26215038 PMC4517641

